# Melatonin plays critical role in mesenchymal stem cell-based regenerative medicine in vitro and in vivo

**DOI:** 10.1186/s13287-018-1114-8

**Published:** 2019-01-11

**Authors:** Chenxia Hu, Lanjuan Li

**Affiliations:** Collaborative Innovation Center for Diagnosis and Treatment of Infectious Diseases, State Key Laboratory for Diagnosis and Treatment of Infectious Diseases, School of Medicine, First Affiliated Hospital, Zhejiang University, Hangzhou Zhejiang, People’s Republic of China

**Keywords:** Melatonin, Mesenchymal stem cell, Regenerative medicine, In vitro, In vivo, Protection, Therapy

## Abstract

Although stem cells have emerged as promising sources for regenerative medicine, there are many potential safety hazards for their clinical application, including tumorigenicity, an availability shortage, senescence, and sensitivity to toxic environments. Mesenchymal stem cells (MSCs) have various advantages compared to other stem cells, including embryonic stem cells (ESCs) and induced pluripotent stem cells (iPSCs); thus, MSCs have been intensely investigated in recent studies. However, they are placed in a harsh environment after isolation and transplantation, and the adverse microenvironment substantially reduces the viability and therapeutic effects of MSCs. Intriguingly, melatonin (MT), which is primarily secreted by the pineal organ, has been found to influence the fate of MSCs during various physiological and pathological processes. In this review, we will focus on the recent progress made regarding the influence of MT on stem cell biology and its implications for regenerative medicine. In addition, several biomaterials have been proven to significantly improve the protective effects of MT on MSCs by controlling the release of MT. Collectively, MT will be a promising agent for enhancing MSC activities and the regenerative capacity via the regulation of reactive oxygen species (ROS) generation and the release of immune factors in regenerative medicine.

## Background

Recently, mesenchymal stem cells (MSCs) have emerged as promising sources for restoring tissue and organ function; however, there are many potential safety hazards for their clinical application, including an availability shortage, senescence, sensitivity to toxic environments, and potential tumorigenicity. Moreover, heterogeneity exists in the isolation and cultivation procedures among laboratories, and the definition of MSCs remains unclear according to current comments from Sipp et al. [1]. Herein, we termed MSCs according to the criteria from the International Society for Cellular Therapy (ISCT). (i) MSCs must be plastic-adherent when maintained in standard culture conditions. (ii) MSCs must express CD105, CD73, and CD90 and lack expression of CD45, CD34, CD14, or CD11b; CD79alpha or CD19; and HLA-DR surface markers. (iii) MSCs must differentiate in vitro into osteocytes, chondrocytes, and adipocytes [2]. Although the nature and functions of MSCs remain unclear, nonclonal stromal cultures obtained from the bone marrow and other tissues that contain a subpopulation of stem cells are currently serving as sources of putative MSCs for therapeutic purposes, largely due to findings that they might be effective in the treatment of several diseases [3]. In addition, MSC-based treatment is powerful and can regenerate organ function through the secretion of cytokines and other anti-inflammatory mechanisms [4]. However, various adverse factors in vitro and in vivo will reduce the stemness of MSCs and commonly hinder the differentiation process of MSCs. Once MSCs are isolated from their original tissues or organs, they rapidly lose their vitality because of inappropriate ex vivo conditions, and shortened telomerase activity and disturbance of the secretome also disturb their morphology [5]. Long-term in vitro culture leads to a decreased colony-forming capacity, reduced proliferation ability, unstable cellular DNA, and a shortened cell lifespan [6, 7]. A sufficient number of active MSCs can compensate for missing organ functions induced by wound, ischemia, drugs, high glucose, sepsis, etc. [8–12]; however, the in vitro life span of MSCs is limited by the harsh microenvironment, which thus leads to an insufficient cell source [6]. On the other hand, transplanted MSCs can secrete beneficial cytokines within the injured area, while more than 80–90% of implanted cells are sentenced to apoptosis or death 72 h after injection by the harsh microenvironment in vivo [13]. Moreover, transplantation of MSCs decreases their survival and proliferation rates because the low blood supply and collagen density of tissue cannot provide sufficient nutrition or growth factors; concurrently, oxidative stress and chronic inflammation are substantially increased and impair MSC activities in vivo [14].

**Fig. 1:**
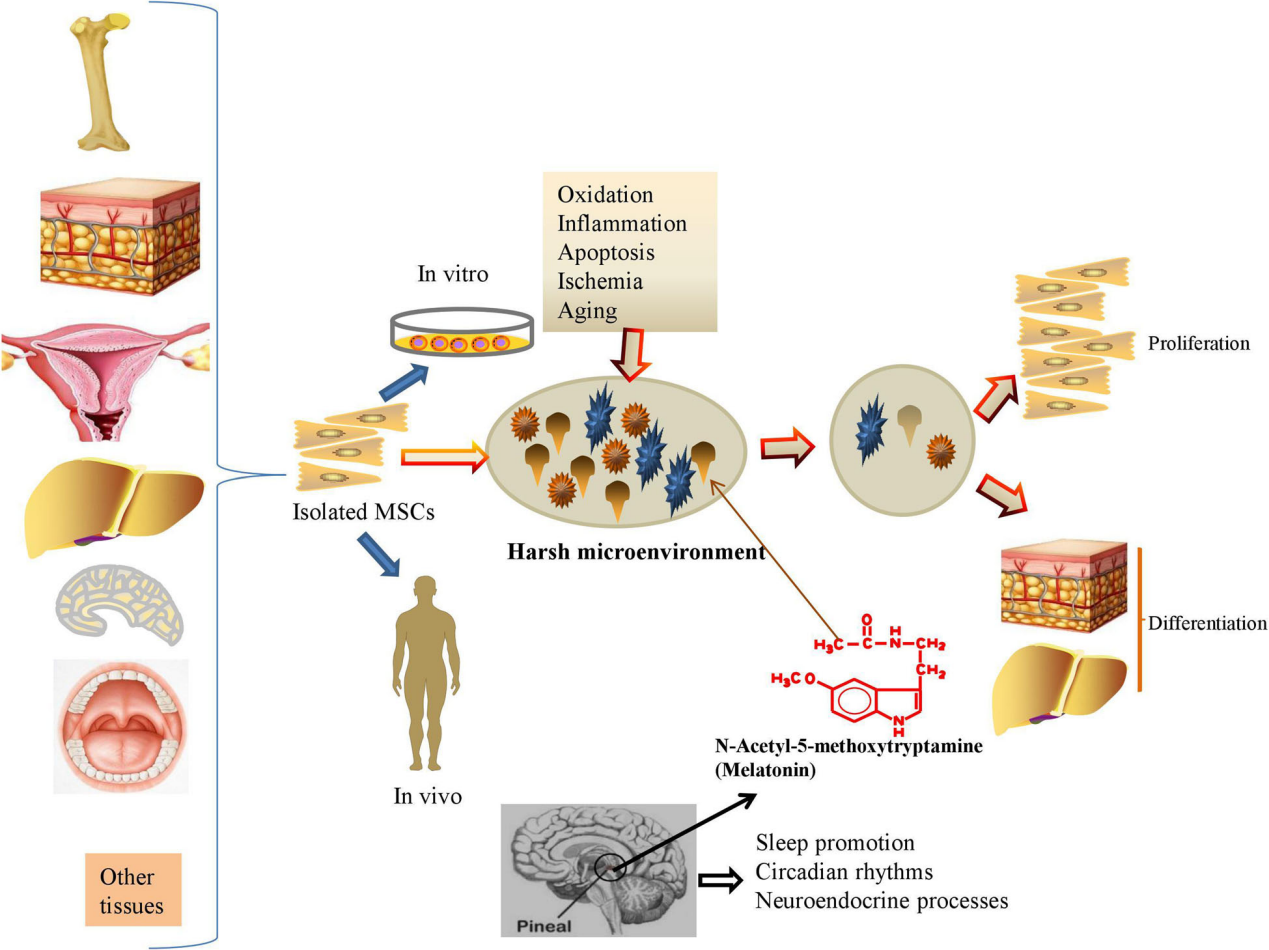
MT has emerged as a novel and potential modulator of MSC fate in vitro and in vivo

Under physiological conditions, MSCs will undoubtedly produce basal reactive oxygen species (ROS) to maintain cell proliferation and differentiation, while incomplete oxidation of oxygen in vitro and in vivo results in excessive production of ROS and DNA damage in MSCs and impairs the normal function of MSCs via multiple apoptosis-related pathways [15, 16]. Extremely high ROS levels generated by hydrogen peroxide (H_2_O_2_), hydroxyl radicals, and superoxide anion will induce oxidative stress in MSCs [17]. The excessive ROS induced by a stressful microenvironment impairs the self-renewal, proliferation, and differentiation capacity of MSCs [18]; moreover, aging significantly upregulates the level of oxidative stress and thus limits the quantity and quality of MSC daughter cells [19]. Recently, melatonin (MT) has been isolated from the pineal organ and shown to participate in regulating multiple physiological functions, including sleep promotion, circadian rhythms, and neuroendocrine processes [20, 21]. The administration of MT substantially improves the manipulation advantages of MSCs ex vivo and in vivo; in general, MT serves as a component of the homeostatic and cell-protective agents, which protect MSCs from oxidation, inflammation, apoptosis, ischemia, and aging for regulating MSC differentiation and protection in different organs and tissues [22, 23]. The antioxidant capacity of MSCs could be particularly relevant in the case of immune and inflammatory components. Importantly, MT is involved in the detoxification of ROS and free radical intermediates because it plays a critical role in the release of antioxidant enzymes, including catalase, glutathione reductase, superoxide dismutase, and glutathione peroxidase [24, 25]. In this article, we review the current literature on the regulatory effects of MT on MSCs undergoing oxidative stress and differentiation; we also discuss its protective effects to improve the therapeutic potential of MSCs in different disease models. In addition to general treatment, several biomaterials emerge for controlling the release of MT, which significantly improves the protective effects of MT on MSCs. Thus, MT has emerged as a novel and potential modulator of MSC fate in vitro and in vivo (Fig. 1). In the near future, MT will be a promising agent for controlling MSC fate via the regulation of ROS generation and release of immune factors in regenerative medicine.

### MT and related agents are promising factors that participate in MSC biology

Mammals secrete a low level of MT during the daytime, and the basal level is lower than 10 pg/mL, while the level of MT is adjusted to higher values (up to 120 pg/mL) at night [26]. MT is not only a hormone secreted by the pineal gland but can also be synthesized by other tissues, including the retina, harderian gland, gastrointestinal tract, testes, and lymphocytes [27]. MT is hypothesized to originate from the mitochondria of eukaryotic cells [28, 29]; it has been demonstrated that the MT concentration is highest in the cell membrane, followed by the mitochondria, nucleus, and cytosol, at the cellular level [30]. Given that mitochondria are the major target of free radicals and the primary intracellular ROS-producing organelle, the lipophilicity of MT is beneficial for exerting its protective effects in vivo [31]. Due to its detoxification and anti-inflammatory properties, MT rescues mitochondrial dysfunction [32], cellular senescence [33], and aging [34, 35]. With respect to energy metabolism, MT significantly upregulates the level of mitochondrial respiration and ATP production, while it inhibits mitochondrial permeability transition pores through the activation of copper, zinc superoxide dismutase (CuZnSOD) within the intermembrane space [29, 36, 37].

Although MT effectively exerts broad free radical scavenging activity without receptors in vivo, the antioxidant activity of MT also requires assistance from specific receptors [38, 39]. Two specific G-protein-coupled receptors, MT1 and MT2 in mammals, bind calmodulin and consequently activate nitric oxide synthase (NOS) [40] and participate in regulating the functions of MSCs [31, 41]. Aside from these two main receptors, MT also interacts with other receptors, including the nonmammalian MT receptor subtype MT3, the nuclear receptor ROR/RZR, and calmodulin [42, 43]. However, the detailed expression level of MT receptors should be determined according to the stem cell type and the physiological status of stem cells. For example, MT stimulates MT1 in cultured placenta-derived MSCs, but not MT2, to increase the cell proliferation and survival rate and further enhance the degree of neuronal differentiation [44]. MSCs isolated from adolescent idiopathic scoliosis patients express lower levels of MT2 and demonstrate lower expression levels of osteogenic and chondrogenic markers than MSCs derived from control healthy individuals after MT treatment [45]. Thus, the MT receptor-dependent pathway also plays a critical role in enhancing MSC stemness and differentiation in regenerative medicine.

MT and its derivatives scavenge free radicals generated from aerobic metabolism and oxidative stress via single-electron transfer, hydrogen transfer, and radical adduct formation [46, 47]. MT can be metabolized into several derivatives, including N1-acetyl-N2-formyl-5-methoxykynuramine (AFMK), N1-acetyl-5-methoxykynuramine (AMK), 6-hydroxymelatonin, 2-hydroxymelatonin, 3-hydroxymelatonin, and cyclic 3-hydroxymelatonin [47, 48]. In addition, special receptor antagonists and agonists neutralize the effects of MT on MSCs, which further helps us untangle the underlying protective mechanisms. Luzindole, which serves as a common nonselective antagonist of MT receptors, activates the MEK/ERK pathway to antagonize the anti-adipogenesis effect of MT on MSCs [49] and blocks the MT-mediated anti-senescence effect [9]. Under the stimulation of MT receptor antagonists, including luzindole and 4P-PDOT, an MT-induced increase in alkaline phosphatase (ALP) activity is highly inhibited, and MT-promoted MSC osteogenesis is thus decreased [50]. Multiple synthetic agonists, including TIK-301, piromelatine, GG-012, AH-001, AH-017, agomelatine, ramelteon, GR 196429, MA-2, tasimelteon, UCM765, and UCM924, regulate circadian rhythms [51]; however, whether these agonists cooperate with MT to protect MSCs from injury remains unknown.

### MT can exert different effects on MSCs in vitro

As the microenvironment is very important to MSCs under in vitro culture, the MSC fate is finely regulated by the surrounding mechanical and molecular signals; thus, MT may act as a protective agent and participate in the regulation of MSC fate. Although ROS act as dangerous toxicants under pathological conditions, MT upregulates CuZnSOD and manganese superoxide dismutase (MnSOD) expression levels and downregulates Bax expression, consequently improving MSC activities, and it reduces ROS production in a dose-dependent manner [52]. MT is an effective agent to alleviate the apoptotic factors for protecting MSCs from injury, while it also acts as an inhibitory agent or a promotive agent according to the MSC differentiation fate (Table 1).

**Table 1:** MT is an effective agent to alleviate the apoptotic factors for protecting MSCs from injury, while it also acts as an inhibitory agent or a promotive agent according to MSC differentiation fate

Concentration	Time point	MSC resource	Effect	Mechanism	Ref
10 nM, 1 μM, 100 μM	Cotreatment	Bone marrow	No effect on the proliferation of MSCs, inhibits adipogenic differentiation of MSCs, enhances MSC osteogenic differentiation	PPARc expression↓, Runx2 expression↑	[49]
50 nM	Cotreatment	Adipose	Increases the ALP expression	MT2 receptor↑, MEK/ERK (1/2) ↑	[50]
10 nM, 1 μM, 100 μM	Cotreatment	Synovium	Improves the proliferation of MSCs, protects cell viability in the presence of IL-1β, promotes MSC osteogenic differentiation when exposed to IL-1β	SOD↑	[52]
1 μM, 10 μM, 100 μM	Cotreatment	Adipose	Reduces the senescent progress and abnormal activation of autophagy	AKT↑, ROS↓	[53]
50 μM, 100 μM	Post-treatment	Bone marrow	Reduces cell death of MSCs	AMPK↑, acetyl-CoA carboxylase↑	[54]
1 μM	Pretreatment	Adipose	Enhances MSC proliferation and self-renewal and diminishes the extent of MSC apoptosis	PrPC-dependent pathway↑	[55]
10–300 nM	Pretreatment	Bone marrow	Protects against hypoxia/serum deprivation-induced injury	ERK1/2↑	[56]
5 μM	Pretreatment	Bone marrow	Prevents MSC apoptosis	Mitogenic factors↑	[57]
10 nM, 1 μM, 100 μM	Post-treatment	Bone marrow	Reverses H_2_O_2_-induced senescence	P38MAPK↓, p16INK4α↓, SIRT1↑	[59]
10 nM, 1 μM, 100 μM	Post-treatment	Bone marrow	Restores the impaired differentiation ability of MSCs induced by H_2_O_2_	SIRT1↑	[59]
10 nM, 1 μM, 100 μM	Pretreatment	Bone marrow	Maintains the morphology, viability, and osteogenic differentiation ability of MSCs	ROS↓, p53/ERK/p38↓	[61]
50 nM	Cotreatment	Bone marrow	Inhibits adipogenic differentiation of MSCs at the early stage of adipogenic differentiation	ROS↓, phosphorylating ERK/GSK-3β↓	[62]
50 nM	Cotreatment	Bone marrow	Promotes chondrogenic differentiation of MSCs	MT receptor-dependent pathway↑	[63]
50 nM	Cotreatment	Bone marrow	Restores the pellet size and matrix accumulation, upregulates chondrogenic differentiation of MSCs, reduces cell apoptosis during the whole chondrogenesis	IL-1β-induced activation of NF-κB signaling↓	[64]
1 μM	Cotreatment	Synovium	Rescues the IL-1β and TNF-α impaired chondrogenesis of MSCs	ROS↓, SOD↑, MMPs↓	[65]
10 μM, 50 μM, 100 μM, 200 μM	Pretreatment	Adipose	Rescues MSCs from cell death induced by oxidative stress; 100 μM of MT confers greater cytoprotection on MSCs than 200 μM	ROS↓, P38MAPK↓, harmful inflammatory cytokines↓	

#### MT serves as a protective agent, promotive agent, or inhibitory agent in MSC physiological and pathological processes in vitro

*p*-Cresol was found to be substantially higher in chronic kidney failure patients than in healthy individuals; it also reduced the proliferation of MSCs in a dose-dependent manner, while pretreatment with MT on MSCs significantly reduced the senescent progress and abnormal activation of autophagy by activating the AKT signaling pathway and inhibiting ROS accumulation [53]. Although tissue-engineered heart valves serve as effective biological materials for repairing congenital cardiac valve diseases, the high flow shear stress significantly reduces the therapeutic effects of MSCs, while MT significantly reduces the cell death of MSCs under a shear stress condition in a concentration-dependent manner by upregulating the phosphorylation of AMPK and acetyl-CoA carboxylase [54]. MT pretreatment enhanced MSC proliferation and self-renewal and diminished the extent of MSC apoptosis in oxidative stress conditions in a prion protein (PrP^C^)-dependent manner [55]. MT pretreatment upregulates p38MAPK and extracellular signal-regulated kinase 1 and 2 (ERK1/2) phosphorylation levels in MSCs and thus protects against hypoxia/serum deprivation-induced injury [56]. Pretreatment with MT decreases H_2_O_2_-induced apoptosis by attenuating ROS generation; partially inhibiting the activation of p38 mitogen-activated protein kinase (P38MAPK) signaling molecule; increasing the release of mitogenic factors, including basic fibroblast growth factor (b-FGF) and hepatocyte growth factor (HGF); and reducing the production of harmful inflammatory cytokines, including tumor necrosis factor (TNF)-α and interleukin (IL)-6 [57, 58]. Zhou et al. demonstrated that pretreatment with MT does not decrease H_2_O_2_-induced premature senescence, while treatment with MT subsequent to H_2_O_2_ exposure effectively reversed the premature senescence in a dose-dependent manner by downregulating p38MAPK phosphorylation and senescence-associated protein p16INK4α and increasing the expression level of sirtuin 1 deacetylase (SIRT1) [59]. To this end, the time point of MT administration is also important for the precise protective effects on MSCs in vitro.

#### MT promotes MSC osteogenesis and chondrogenesis and inhibits adipogenesis

In addition, MT can not only influence MSC activities ex vivo but also influence the differentiation abilities, particularly osteogenesis. Radio et al. first showed that the addition of MT at a concentration of 50 nM into osteogenic medium increased the ALP expression level to a certain extent in MSCs via upregulating the MT2 receptor and the MEK/ERK (1/2) signaling pathway [50]. Moreover, MSCs were cultured in osteogenic differentiation medium with IL-1β for 21 days, and 1 μM MT significantly increased the levels of type I collagen, ALP, and osteocalcin, while 100 μM MT treatment yielded the highest level of osteopontin [52]. The addition time point of MT exposure will certainly influence the final differentiation fate of MSCs, as it was demonstrated that the addition of MT at the initiation stage or the end stage of osteogenesis increases the rate of mature cells, while addition at the intermediate stage does not influence the outcome [60]. Moreover, H_2_O_2_-induced premature senescence and an iron overload microenvironment resulted in a decreased osteogenic differentiation potential, and MT restored the impaired differentiation ability of MSCs by upregulating the SIRT1-dependent pathway and blocking p53/ERK/p38 signaling, respectively [59, 61].

Despite the promotive effects on MSC osteogenesis, MT can exert completely opposite effects on MSC adipogenesis in vitro. MT enhances MSC osteogenesis by upregulating the expression levels of RUNX2, osteopontin, and osteocalcin and inhibits adipogenesis in the same population by suppressing peroxisome proliferator-activated receptor gamma (PPARγ), leptin, lipoprotein lipase (LPL), adiponectin, and adipocyte protein 2 expression [49]. In addition, MT inhibits the adipogenic differentiation of MSCs by regulating the expression level of C/EBPβ at the early stage of adipogenic differentiation, consequently reducing cyclic adenosine monophosphate synthesis by reducing ROS generation and phosphorylating ERK/GSK-3β [62].

Go et al. also proposed that MT promoted chondrogenic differentiation of MSCs because they observed higher levels of chondrogenic markers and more cartilage tissue and collagen isotypes in the MT group than in the control group partially through MT receptors [63]. While IL-1β clearly impaired the chondrogenesis of human MSCs, cotreatment with MT significantly restored the pellet size and matrix accumulation, upregulated the expression levels of chondrogenic markers, maintained the metabolic balance, and reduced cell apoptosis during the whole chondrogenesis by attenuating the IL-1β-induced activation of NF-κB signaling [64]. Similarly, proinflammatory cytokines, including IL-1β and TNF-α, impair the chondrogenesis of MSCs and resulted in cartilage degradation; however, it can be rescued by MT treatment because it can significantly decrease the ROS level, preserve SOD, and suppress the expression levels of matrix metalloproteinases (MMPs) [65].

In this scenario, MT seems to not only drive the commitment and differentiation of MSCs under specific culture conditions but also repair impaired differentiation induced by a harsh microenvironment through regulating paracrine signaling pathways and controlling the ROS level. Moreover, it is necessary to explore the effects of MT in vitro on other differentiation directions in addition to osteogenesis, chondrogenesis, and adipogenesis.

### MT significantly improves the therapeutic effects of MSCs in vivo

**Fig. 2:**
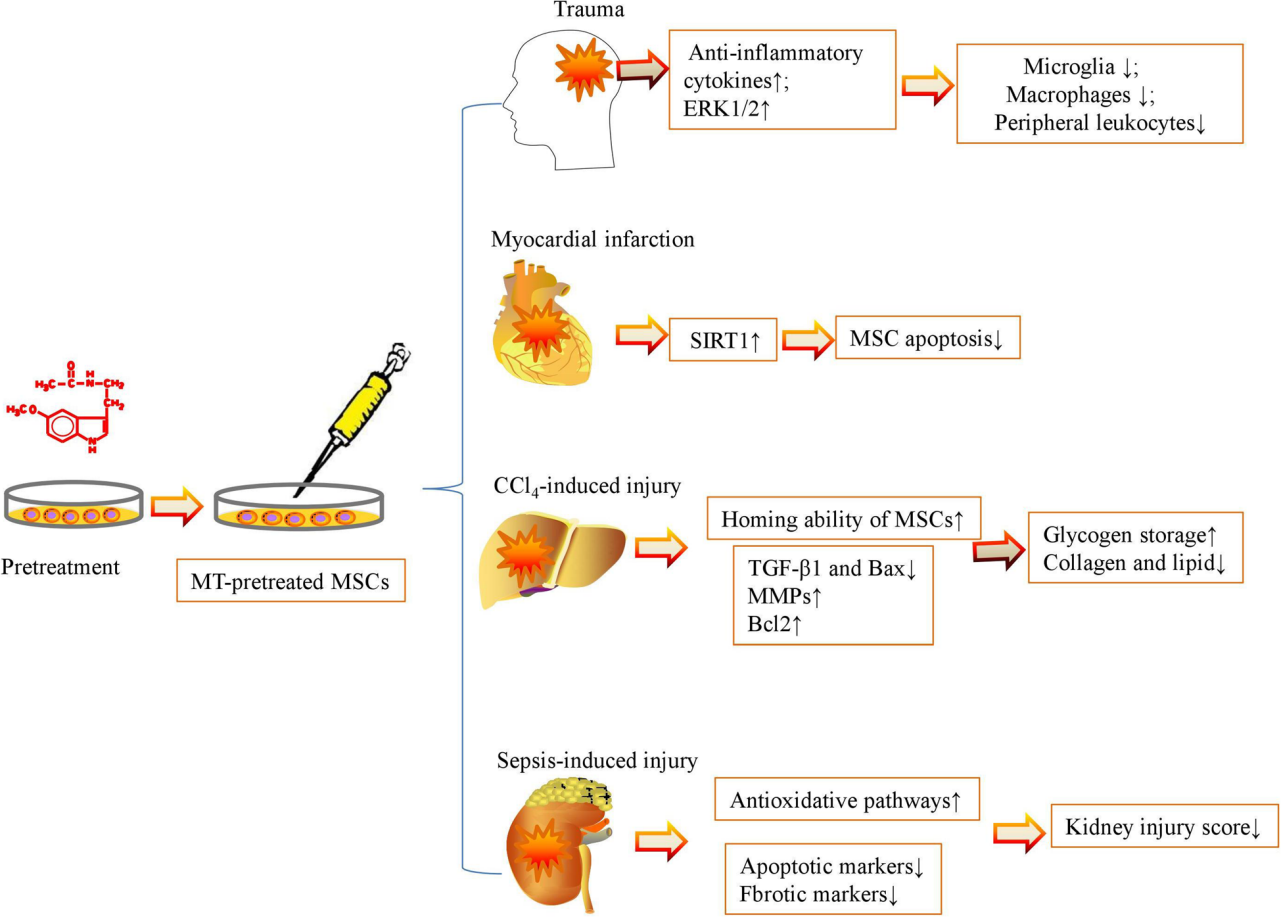
MT increases MSC activities to repair functions of large organs, such as the brain, heart, liver, and kidney

Harsh in vivo microenvironments effectively weaken the therapeutic effect of MSC-based treatment. In recent years, MT has also emerged as an effective agent for increasing the survival rate of MSCs and provoking a synergistic effect to rescue the function of multiple organs by alleviating inflammation, apoptosis, and oxidative stress (Table 2). MT is widely used to improve the homing ability and decrease the apoptosis rate of MSCs after transplantation in large organs, such as the brain, heart, liver, and kidney (Fig. 2), and the other organs targeted for tissue engineering include the skin, pancreas, small bowel, and limb.

**Table 2:** MT enhances MSC activity to rescue the function of multiple organs by alleviating inflammation, apoptosis, and oxidative stress

Animal	Disease	Dose	MSC resource	Effect	Mechanism	Ref
C57BL/6a mice	Myocardial infarction	5 μM	Adipose	Heart function↑	SIRT1↑	[14]
Male ICR mice	Skin wound	1 μM	Umbilical cord blood	Wound closure↑; granulation↑; re-epithelialization↑	MT2-mediated pathway↑	[8]
Adult male Sprague Dawley rats	Focal cerebral ischemia	0.05 to 1 mM	Bone marrow	Brain infarction↓; neurobehavioral outcomes↑	ERK1/2 ↑	[9]
Adult male Sprague Dawley rats	Liver fibrosis	5 μM	Bone marrow	Glycogen storage↑; collagen and lipid accumulation↓	TGF-β1 and Bax/Bcl2↓	[10]
Male albino rats	Diabetes	10 mg/kg for 8 weeks	Bone marrow	Structural and functional efficiency of β-cells in the pancreas↑	Antioxidant↑	[11]
Adult male Sprague Dawley rats	Acute kidney injury	After the procedure, 20 mg/kg at 30 min, 50 mg/kg at 6 h and 18 h	Adipose	Sepsis induced acute kidney injury↓	Inflammation↓, inappropriate immune response↓, ROS generation↓, oxidative stress↓	[12]
Male BALB/c nude mice	Limb ischemia	After procedure, 20 mg/kg/day for 28 days	Adipose	MSC functionality↑; neovascularization↑	PrP^C^ expression↑	[55]
Adult male Sprague Dawley rats	Small bowel ischemia reperfusion	After the procedure: 20 mg/kg, 50 mg/kg at 6 h and 18 h	Adipose	Ischemia reperfusion injury↓	Inflammation↓, oxidative stress↓, apoptosis↓, mitochondrial damage↓, DNA damage↓, antioxidant enzymes↑	[66]
Adult male Sprague Dawley rats	Acute interstitial cystitis	After the procedure, 20 mg/kg at 30 min, 50 mg/kg at 6 h and 18 h	Adipose	Cyclophosphamide-induced acute interstitial cystitis↓	Antioxidants↑	[67]
Adult male Wistar strain Albino rats	Diabetic nephropathy	5 μM	Bone marrow	Kidney functions↑	Pathogenic factors↓, autophagy↑	[68]

MSCs are widely used in the treatment of wound-induced injury and ischemia injury in various tissues, and MT has been applied to improve the therapeutic effects of MSCs via anti-inflammation and antioxidative stress. Pretreatment with MT improved the MSC motility and enhanced the granulation and re-epithelialization of transplanted cells in a mouse skin excisional wound model via an MT2-mediated pathway. Thus, although MT induced the phosphorylation of focal adhesion kinase (FAK) and paxillin, increased the levels of active Cdc42 and Arp2/3, and stimulated cytoskeletal reorganization-related proteins, such as profilin-1, cofilin-1, and F-actin, in MSCs, a lack of MT2 in MSCs during a mouse skin transplantation experiment resulted in impaired wound healing and less engraftment of MSCs at the wound site [8]. In the ischemic brain, MT pretreatment substantially increased the survival of MSCs in vitro and reduced their apoptosis after transplantation in vivo. The MT-pretreated MSCs significantly upregulated angiogenesis, neurogenesis, and the expression of vascular endothelial growth factor (VEGF), which thus reduced brain infarction and improved neurobehavioral outcomes in the animal models via ERK1/2 signaling pathway activation [9]. Similarly, MT effectively improved the survival rate of MSCs and provoked MSCs to restore heart function by activating the SIRT1 signaling pathway and alleviating inflammation, apoptosis, and oxidative stress in the infarcted heart [14]. The combination of MSCs and MT decreased the circulating levels of TNF-α, MPO, LyG6+ cells, CD68+ cells, and WBC count; the gut permeability; and the ischemic mucosal damage score in small bowel ischemia-reperfusion (SBIR) rats [66]. After transplantation into a hind-limb ischemia murine model, MT-pretreated MSCs decreased the number of local apoptotic cells and improved the blood flow perfusion, limb salvage, and vessel regeneration to restore limb function through the upregulation of PrP^C^ expression [55]. The authors suggested that-MT pretreated MSCs would provide a powerful tool for promoting neovascularization in patients suffering from ischemic diseases.

In animal studies, various toxicants have been applied to mimic diseases in humans to further clarify the underling mechanisms and potential effective treatments. To improve the therapeutic effects of MSCs in the treatment of carbon tetrachloride (CCl_4_)-induced liver injury, MT-pretreated MSCs significantly increased the level of glycogen storage and decreased the level of collagen and lipid accumulation in the injured liver after MSC transplantation [10]. Moreover, although MT did not exert effects on collagen deposition, tissue disruption, and fatty accumulation, it significantly downregulated the expressions of TGF-β1 and Bax and upregulated the expressions of MMPs and Bcl2 in the injured liver [10]. The combined regimen of MT and MSCs was superior than either alone in protecting against cyclophosphamide-induced acute interstitial cystitis (AIC) rats as it significantly reduced the severity of hematuria, release of inflammatory cytokines, glycosaminoglycan, oxidized protein, and ROS in the bladder tissue and improved the integrity of the epithelial layer and area of collagen deposition [67].

As diabetes is a type of chronic disease with a high mortality, it also brings out multiple complications that influence the survival quality of humans. Thus, MSC transplantation is also taken into account for one of the effective treatments for diabetes. The cotreatment of MSCs and MT significantly improved the levels of glucose, insulin, total antioxidant, and malondialdehyde and reduced the number of damaged β-cells in diabetic rats compared with the MSC group alone [11]. Diabetic nephropathy (DN) is one of the microvascular complications of diabetes that may progress to a serious end-stage renal failure. Pretreatment with MT also well-maintained the kidney functions of DN pathogenesis accompanied by higher levels of SOD-1 and Beclin-1 and a lower level of TGF-β in the kidney tissue than in the MSC group [68].

Sepsis is a type of systemic inflammatory response syndrome in response to documented infection in the blood, sputum, urine, or normally sterile body fluid. Sepsis can progress to multiple organ dysfunction and death [69]. Intriguingly, Chen et al. used the combination of MT with apoptotic adipose-derived MSCs and found that the combination provided additional benefits in ameliorating sepsis-induced acute kidney injury through the activation of antioxidative pathways, inhibition of the inflammatory response, and reduction of apoptosis and fibrosis [12]. These findings raise the potential strategy for treating sepsis-induced injury in other organs via the combination of MT and MSCs.

### Biomaterials for enhancing the protective effect of MT on MSCs

Currently, various preconditioning strategies are applied in stem cell therapy to provide resistance against injury and enhance the antioxidant capability after MSC implantation. In addition to interaction with growth factors derived from MSCs, the interaction of MT with biomaterials can vigorously improve the therapeutic effects of regenerative medicine. Zhang et al. encapsulated MT into poly (lactic-co-glycolic acid) (PLGA) microspheres (PLGA-MEL-MS) for the sustained release of MT; PLGA-MEL-MS had no apparent effect on the proliferation of human MSCs, while it enhanced the expression levels of runx2, osteopontin, and osteocalcin and enhanced the calcium deposit of human MSCs compared to the control group [70]. To enhance the osteoinductive and osteoconductive properties of calcium aluminate (CA) scaffolds, a system that consists of MT and CA scaffolds has been demonstrated to enhance the adhesion, viability, and proliferation of human osteoblast cells but not that of NIH3T3 fibroblasts; it also promoted the osteogenesis of MSCs into osteoblasts over 14 days [71]. Given that MT is an excellent agent to regulate the fate of MSCs, Lai et al. constructed a device, namely, Chi/Gel multilayer-coated melatonin-loaded TiO_2_ nanotube substrates, to control the sustained release of melatonin; they found that the sustained release of melatonin could improve the cell proliferation rate and osteoblastic differentiation of MSCs [72]. Nano drug delivery carriers are easy to control the release of drug with polymer membranes, which thus improves the bioavailability of drugs and reduces the required dosage and side effects of drugs. Poly(lactide-co-glycolide)-monomethoxy-poly-(polyethylene glycol) (PLGA-mPEG) nanoparticles were used to encapsulate melatonin and generated melatonin nanoparticles (Mel-NPs). Ma et al. found that Mel-NPs reduced the formation of the p53-cyclophilin D complex, prevented mitochondrial permeability transition pores from opening, improved the MSC survival rates, and rescued MSCs from hypoxia/reoxygenation injury in a controlled manner [73]. Pretreatment with Mel-NPs also improved the MSC survival rates compared with MT in rat myocardial infarction areas and improved the therapeutic effects of MSCs [73]. Thus, the combination of Mel-NPs and stem cell transplantation may be a promising strategy for myocardial infarction therapy. Moreover, the combination of nanomaterial and other new biomaterial with MT may be a novel preconditioning method to improve the efficiency of MSC transplantation.

## Conclusion

The excessive ROS during proliferation and differentiation in vitro and the injured microenvironment in vivo significantly reduce the therapeutic effects of MSCs for repairing injury in vivo. MT is a hormone with myriad biological functions, including but not limited to anti-inflammation, oncostatis, circadian and endocrine rhythm regulation, and tumor inhibition. Current evidence has demonstrated that MT can reduce the release of inflammatory cytokines, enhance the proliferation capacity of MSCs, and eliminate the apoptosis of MSCs in vitro and in vivo. Furthermore, as MT is progressively becoming an attractive agent for the regeneration of various organs or tissues, we highlighted that MT regulated the expression of ROS-generating and antioxidant genes as upstream events in its protective and anti-apoptotic mechanisms of MSCs according to the current evidence. In addition, MT has been proven to promote MSC osteogenesis and chondrogenesis and inhibit adipogenesis, and it may be further added as an effective agent in the differentiation medium. After the optimal concentration and optimal administration time point are determined, the protective effects of MT on MSCs can be improved in the near future. It is worth noting that high MT concentrations may harm the epigenetic and genetic stability of MSCs. We believe that the clarification of the underlying mechanisms for eliminating injury in more disease models or patients will shed light on the protective effects of MT on MSC-based cell and tissue engineering.

## Abbreviations

AFMK: N1-acetyl-N2-formyl-5-methoxykynuramine
AIC: Acute interstitial cystitis
AMK: N1-acetyl-5-methoxykynuramine
b-FGF: Basic fibroblast growth factor
BMP2: Bone morphogenetic protein 2
CA: Calcium aluminate
CCl_4_: Carbon tetrachloride
CuZnSOD: Copper, zinc superoxide dismutase
DN: Diabetic nephropathy
ERK1/2: Extracellular signal-regulated kinase 1 and 2
FAK: Focal adhesion kinase
GAG: Glycosaminoglycan
H2O2: Hydrogen peroxide
HGF: Hepatocyte growth factor
IL: Interleukin
ISCT: International Society for Cellular Therapy
LPL: Lipoprotein lipase
Mel-NPs: Melatonin nanoparticles
MMPs: Matrix metalloproteinases
MnSOD: Manganese superoxide dismutase
MSCs: Mesenchymal stem cells
MT: Melatonin
NOS: Nitric oxide synthase
P38MAPK: P38 mitogen-activated protein kinase
PLGA: Poly (lactic-co-glycolic acid)
PLGA-mPEG: Poly(lactide-co-glycolide)-monomethoxy-poly-(polyethylene glycol)
PPARγ: Peroxisome proliferator-activated receptor gamma
PrPC: Prion protein
ROS: Reactive oxygen species
RUNX2: Runt-related transcription factor 2
SBIR: Small bowel ischemia-reperfusion
SIRT1: Sirtuin 1 deacetylase
TNF: Tumor necrosis factor
VEGF: Vascular endothelial growth factor
